# Revisiting definitions of hypervirulent and classical *Klebsiella pneumoniae* through virulence factors and disease phenotype

**DOI:** 10.1038/s43856-026-01613-7

**Published:** 2026-05-27

**Authors:** Alfred Lok-Hang Lee, Carmen Li, Rita WY Ng, Christopher KC Lai, Peter Chi-Wai Yip, Wing Shan Lee, Lap-Tin Ho, Ingrid Yu-Ying Cheung, Viola Chi-Ying Chow, Margaret Ip

**Affiliations:** 1 Department of Microbiology, Prince of Wales Hospital, Sha Tin, Hong Kong, (SAR) China; 2 Department of Microbiology, Faculty of Medicine, The Chinese University of Hong Kong, Sha Tin, Hong Kong, (SAR) China; 3 S. H. Ho Research Centre for Infectious Diseases, The Chinese University of Hong Kong, Sha Tin, Hong Kong (SAR) China; 4 Intensive Care Unit, North District Hospital, Sheung Shui, Hong Kong, (SAR) China

**Keywords:** Infectious-disease epidemiology, Clinical microbiology

## Abstract

**Background:**

Hypervirulent *Klebsiella pneumoniae* (hvKP) has been described as an invasive syndrome of liver abscess and metastatic infection in Asia, and this syndrome is emerging globally.

**Methods:**

This study examined the association between disease phenotypes and genotypic markers of hypervirulence in 273 isolates of *Klebsiella pneumoniae* (KP) collected from invasive infections across three hospitals in Hong Kong between 2019 and 2023. Whole genome sequencing was analysed by Kleborate and Kaptive. Epidemiological background, disease phenotype, virulence genes, and antimicrobial susceptibility were analysed. Genotypically defined hvKP (g-hvKP) were defined as having *iucA*, *iroB*, *rmpA*, *rmpA2*, and *peg*-*344*. Others were defined as classical *Klebsiella pneumoniae* (cKP). Genome-wide association study (GWAS) was performed to identify additional genes associated with hvKP.

**Results:**

The commonest K types are K2 (13.9%) and K1 (10.6%). The commonest ST is ST 23 (8.8%). g-hvKP represents 20.8% of all isolates. The ESBL phenotype is more likely in cKP than g-hvKP (22% *vs* 9%, *p* = 0.04). g-hvKP are more susceptible to cephalosporins (*p* = 0.02) and fluoroquinolones (*p* = 0.01). Among liver abscess cases, 49% of them are c-KP. g-hvKP is associated with neither mortality, disease severity, nor metastatic infection. g-hvKP has a sensitivity of 46% and a specificity of 86% in detecting liver abscesses or metastatic infections. By omitting *rmpA2*, F1 score improves from 0.46 to 0.51. GWAS reveals *cysH3*, *pfeA2*, and *aidA* as additional candidate genes linked to hvKP.

**Conclusion:**

Significant overlap in disease phenotype exists between cKP and g-hvKP. Additional candidate genes linked to hvKP are *cysH3*, *pfeA2*, and *aidA*.

## Plain language summary

The bacterial pathogen *Klebsiella pneumoniae* (Kp) can cause dangerous, invasive infections in hospitals. A subset of infections with a “hypervirulent” form of Kp are particularly dangerous, but exact definitions of what constitutes hypervirulent Kp are still in question. In this study of 273 serious Kp infections, we found that only 1 in 5 cases carried the classic hypervirulent DNA markers. We also found that among the most severe liver damage seen in infection, only half of cases were caused classically defined hypervirulent Kp. The classical DNA-based definition also was not able to predict disease severity. We found non-classical DNA markers among these serious infections, suggesting the old definition of “hypervirulent” Kp needs updating.

## Abstract (web summary)

Lok-Hang Lee et al. reviewed 273 cases of invasive *Klebsiella pneumoniae* (KP) infection and discovered significant overlap in disease phenotype between classical KP (cKP) and genotypic hypervirulent KP(g-hvKP). They then performed a genome-wide association study, revealing *cysH3*, *pfeA2*, and *aidA* as candidate genes for the hypervirulent disease phenotype.

## Introduction

*Klebsiella pneumoniae* is an important pathogen in both community and hospital settings. Globally, *Klebsiella pneumoniae* is an important source and vehicle for antimicrobial resistance in healthcare settings^1^. It is one of the major contributors of Carbapenemase-producing Enterobacterales, carrying carbapenemase genes such as *bla*KPC, *bla*NDM, and *bla*IMP^2^. Commonly, *Klebsiella pneumoniae* causes nosocomial intra-abdominal infection, urinary tract infection, and pneumonia in hospitalised patients with significant comorbidities^3^.

Hypervirulent *Klebsiella pneumoniae* (hvKP) of serotype K1 in Asian countries characteristically belongs to ST23, and is resistant to phagocytosis and serum killing^4^, This is also described as an invasive syndrome across the Asia-Pacific region, classically associated with community-acquired liver abscess, bacteraemia with a tendency for extraintestinal and metastatic spread, with manifestations including pneumonia, endophthalmitis, central nervous system infection, and other metastatic abscess formation^5^. Subsequently, the global spread of hvKP becomes alarming, especially with the accumulation of multidrug resistance^6–9^. The definition of hvKP ranges from clinical definition, i.e., the ability to cause community-onset liver abscess and metastatic infection, to phenotypic markers such as quantitative siderophore production, to genotypic markers such as *peg-344* (putative DMT transporter gene), *iroB* (salmochelin gene), *iucA* (aerobactin gene), *rmpA* (regulator of mucoid phenotype A), and *rmpA2* (regulator of mucoid phenotype A2)^4,10–14^. Of note, a combination of the 5 genomic markers is shown to be predictive of more severe disease and higher early mortality in a large cohort of patients^11^.

In Asia, the genomic epidemiology of *Klebsiella pneumoniae* is highly diverse. Classically, hvKP is considered more susceptible to antibiotics. However, in recent studies, the convergence of virulence markers and antimicrobial resistance markers such as extended-spectrum beta-lactamase (ESBL) or carbapenemase is observed^6,7^. Common sequence types (ST) and K serotypes for hvKP in the region include ST23 (K1), ST65 (K2), and ST86 (K2)^5^.

In this study, we aim to analyse the relationship between the disease phenotype and the genotypic markers of hypervirulence in a cohort of patients with *Klebsiella pneumoniae* bacteraemia. In total, 273 cases of *Klebsiella pneumoniae* (KP) invasive infections and the genomes of the corresponding isolates are studied. There is significant overlap in disease phenotype between classical KP (cKP) and genotypic hypervirulent KP (g-hvKP) and no difference in mortality between the two. Among KP liver abscess, half of the cases are caused by cKP. Genome-wide association study revealed *cysH3*, *pfeA2*, and *aidA* as additional candidate genes for the hypervirulent disease phenotype.

## Materials and methods

### Bacterial isolates

Two hundred and seventy-three isolates of *Klebsiella pneumoniae* from blood and cerebrospinal fluid (CSF) cultures of 265 hospitalised patients of three hospitals in the New Territories East region of Hong Kong, namely Prince of Wales Hospital, North District Hospital, and Shatin Hospital, in the period 1st January 2019 to 31st December 2023, were included in the analysis. For multiple isolates obtained from the same patient, isolates collected within 1 month interval were removed.

Phenotypic antimicrobial susceptibility test through disc diffusion method was performed and interpreted in accordance with Clinical & Laboratory Standards Institute (CLSI) M100 2022 guidelines. Eleven antibiotics were tested, namely amoxicillin-clavulanate, piperacillin-tazobactam, cefuroxime, cefotaxime, cefepime, ertapenem, meropenem, gentamicin, and amikacin. ESBL for all isolated were detected by the combination disc method as described in CLSI M100 2022 Table 3A.

The clinical definition of hvKP was community-onset bacteraemia with liver abscess, meningitis, endophthalmitis, or metastatic infection^4^. Community-onset infection was defined as cases with blood culture collection within 48 hours of hospital admission.

The definition of genotypically defined hvKP (g-hvKP) was the combination of *peg-344, iucA, iroB, rmpA*, and *rmpA2*^10,11^. Other *Klebsiella pneumoniae* isolates were classified as classical *Klebsiella pneumoniae* (cKP).

To ensure the robustness of the findings, a second set of analyses was performed using only the genotypic determinants of hypermucoidy, i.e., the presence of intact *rmpA* and/or *rmpA2*, as an alternative definition of g-hvKP.

### Whole genome sequencing and analysis

DNA was extracted from bacterial isolates, and the library preparation was performed with Twist 96-Plex Library Preparation Kit (Twist Bioscience, CA, USA). Whole genome sequencing was performed on either Illumina HiSeq or Miseq platform. Quality assessment of the genomes and analyses was performed as previously described^2^. Briefly, genome reads were trimmed with trimmomatic and assembled via Spades, while quality control of the strains was performed using Kraken and Quast for contamination and contig sizes. Analyses of *Klebsiella pneumoniae* were done using Kleborate (v2.4.1) and visualised using Kleborate-viz. A whole genome SNP tree was performed using PARSNP with reference genome *Klebsiella pneumoniae* ATCC BAA-1705. Abricate (v1.0.1) was used for the identification of the gene *peg-344*. Visualisation of the tree and metadata was done with iTOL. Genome data were annotated by Prokka (v1.14.6). Pan genome calculation was done by ROARY using a minimum blastp identity cut-off of 90%. Genomes of all isolates were deposited on NCBI GeneBank (accession number: PRJNA1307753).

### Clinical and epidemiological information

Baseline clinical and epidemiological information was retrieved from the hospital electronic database for clinical management, the Clinical Data Analysis and Reporting System (CDARS). CDARS is a territory-wide database that encompasses all clinical data from all public hospitals and clinics in Hong Kong. Public hospitals in Hong Kong provide 80% of all hospital admissions locally, with the proportion of total bed-days reaching 90%^15^. Nearly all patients with critical emergencies are treated by the emergency departments in the public sector in Hong Kong. Patient demographics, clinical manifestations, site(s) of infection, co-morbidities, outcome measures, and date of death (if applicable) were retrieved for analyses.

### Ethical considerations

Clinical ethics for the project was approved by the Joint CUHK-NTEC Clinical Research Ethics Committee (ref. no.: 2019.013). The requirement for patient consent was waived by the ethics board as this was a retrospective study.

### Statistics and reproducibility

For exploratory analysis, categorical variables were compared using the Fisher’s exact test. Continuous variables were compared using the Wilcoxon rank-sum test.

Using the clinical definition of hvKP as the reference standard, the utility of different definitions of g-hvKP was evaluated by sensitivity, specificity, positive predictive value (PPV), negative predictive value (NPV), and F1 score. The F1 score was the key comparator of the classification performance of each genotypic definition.

To identify factors associated with each disease manifestation, logistic regression was used to identify significant predictors. Univariable logistic regression was used, with each clinical, epidemiological, and virulence gene as the independent variable and the disease manifestation as the dependent variable. Independent variables with *p*-values lower than the threshold were included in multivariable logistic regression.

The Cox proportional hazard model was used to analyse time to death. Univariable logistic regression was first used to screen for potential key predictors, which were then entered into the multivariable regression model. 14-day mortality was also investigated with multivariable logistic regression.

Genome-wide association study (GWAS) was performe d to investigate the relationship between the presence/absence of genes and the clinical hvKP phenotype on all isolates. Genes with relative frequency lower than 5% were excluded from the GWAS. Fisher’s exact test was performed using clinical hvKP phenotype and the presence/absence of each gene as the two categorical variables. The Bonferroni correction was applied. The overall family-wise error rate was set at 0.05. Genes with *p*-values smaller than the Bonferroni-corrected *p*-value threshold was considered statistically significant. Manhattan plots were used to illustrate the range of *p*-values obtained from the GWAS and identify significant genes.

Preliminary exploration of additional candidate genes for hypervirulence was performed on 4 sequence collections of *Klebsiella pneumoniae* from NCBI (BioProject accession number: PRJNA395086, PRJNA658369, PRJNA788509, PRJNA1003408)^16–19^. Genome annotation was performed as described above in the section “Whole genome sequencing and analysis”. The respective BioSample metadata, specifically relating to the host condition and the isolation source where available, were also obtained from NCBI. Isolates from blood or other sterile sites were classified as “invasive”, while isolates from other sites were classified as “non-invasive”. The association between the additional candida genes from GWAS and the nature of infection (“invasive” *vs* “non-invasive”) was assessed using chi-squared test.

All analyses were performed using R Statistical Software (v4.4.1; R Core Team 2024). Except for GWAS, *p*-values lower than 0.05 were considered statistically significant.

## Results

### Demographics

A total of 273 consecutive isolates from 265 patients in the study period 2019-2023 were collected, among which 272 were from blood and 1 was from cerebrospinal fluid. Eight patients contributed 2 isolates, and 257 patients contributed 1 isolate. The mean age of the cohort was 72.2 years, and 156 (57.1%) patients were male. The mean age-adjusted Charlson comorbidity index (ACCI) was 5.4. Forty-four (16.1%) patients were elderly care home residents, and 23 cases (8.4%) were hospital-acquired, i.e. onset beyond 48 hours of admission. Thirty-three cases (12.1%) required admission to the intensive care unit (ICU). The 30-day mortality was 25.6%.

There was no statistical difference between patients infected with cKP and g-hvKP in terms of age, gender, percentage of care home residents, hospital-acquired infections, admission to ICU, inotrope use, and mortality at various cut-offs. Patients with g-hvKP infection had a lower Charlson comorbidity index (*p* = 0.05). For the summary of epidemiological information, please refer to Table 1 and Supplementary Table 1.

**Table 1:** Epidemiological, clinical, and genomic comparison of cKP and g-hvKP

Variables	cKP (*n* = 216) Count (%)	g-hvKP (*n* = 57) Count (%)	*p*-values
**Epidemiological characteristics**			
Age in years^a^	72.4 (14.6)	71.4 (15.2)	0.75
Male gender	127 (59%)	29 (51%)	0.30
Hospital-onset infection	20 (9%)	3 (5%)	0.43
Residents of elderly care home	30 (14%)	14 (25%)	0.07
14-day mortality	45 (21%)	9 (16%)	0.46
30-day mortality	58 (27%)	12 (21%)	0.40
1-year mortality	107 (50%)	27 (47%)	0.88
**Clinical types of infection**			
Metastatic infection (2 or more sites involved)	12 (6%)	9 (19%)	0.02
Liver abscess	23 (11%)	24 (42%)	<0.01
Biliary infection	74 (34%)	11 (19%)	0.04
Other intra-abdominal infection	12 (6%)	1 (2%)	0.31
Pneumonia	38 (18%)	13 (23%)	0.44
Urinary tract infection	52 (24%)	9 (16%)	0.21
Bone and joint infection	4 (2%)	1 (2%)	1.00
Central nervous system infection	1 (0%)	1 (2%)	0.37
Endophthalmitis	1 (0%)	2 (4%)	0.11
**Severity of infection and clinical management**			
APACHE II score^a^	26.7 (11.2)	22.7 (10.9)	0.36
Intensive care unit admission	22 (10%)	11 (19%)	0.07
Inotrope use	43 (20%)	17 (30%)	0.15
Effective antibiotics within 48 hours of onset	194 (90%)	51 (89%)	1.00
**Medical comorbidities**			
Age-adjusted Charlson comorbidity index^a^	5.8 (3)	4.9 (3.1)	0.05
Diabetes mellitus	132 (61%)	34 (60%)	0.88
Pre-diabetes	41 (19%)	13 (23%)	0.58
Immunosuppressant use	43 (20%)	8 (14%)	0.35
Colorectal carcinoma	14 (6%)	3 (5%)	1.00
Hepatobiliary or pancreatic malignancy	42 (19%)	3 (5%)	0.01
Other solid organ malignancy	14 (6%)	3 (5%)	1.00
Haematological malignancy	11 (5%)	0 (0%)	0.13
Cirrhosis	12 (6%)	4 (7%)	0.75
Gallstones	52 (24%)	9 (16%)	0.21
Myocardial infarction	11 (5%)	3 (5%)	1.00
Congestive heart failure	18 (8%)	6 (11%)	0.60
Peripheral vascular disease	3 (1%)	0 (0%)	1.00
Cerebrovascular disease	50 (23%)	12 (21%)	0.86
Dementia	8 (4%)	1 (2%)	0.69
Chronic pulmonary disease	19 (9%)	3 (5%)	0.58
Rheumatic disease	0 (0%)	0 (0%)	1.00
Peptic ulcer disease	22 (10%)	4 (7%)	0.62
Renal disease	29 (13%)	4 (7%)	0.25
Hypertension	84 (39%)	23 (40%)	0.88
**Genotypic epidemiology**			
ST23	4 (2%)	20 (35%)	<0.01
ST65	6 (3%)	7 (12%)	0.01
ST37	12 (6%)	0 (0%)	0.08
K1 serotype	9 (4%)	20 (35%)	<0.01
K2 serotype	24 (11%)	14 (25%)	0.02
O1O2v1 serotype	74 (34%)	16 (28%)	0.43
O1O2v2 serotype	70 (32%)	31 (54%)	<0.01
**Genotypic markers of virulence**			
*ybt*	84 (39%)	42 (74%)	<0.01
*clb*	13 (6%)	24 (42%)	<0.01
*iucA*	43 (20%)	57 (100%)	<0.01
*iroB*	44 (20%)	57 (100%)	<0.01
*rmpA*	44 (20%)	57 (100%)	<0.01
*rmpA2*	1 (0%)	57 (100%)	<0.01
*peg-344*	44 (20%)	57 (100%)	<0.01
KpVP-1 virulence plasmid	19 (8%)	56 (98%)	<0.01
KpVP-2 virulence plasmid	6 (3%)	0 (0%)	0.59
**Genotypic markers of resistance**			
Number of resistance genes^a^	4.1 (5.0)	0.7 (1.8)	<0.01
*bla*LAP2	24 (11%)	0 (0%)	<0.01
*bla*TEM1Dv1	38 (18%)	2 (4%)	0.01
*bla*OXA	23 (11%)	0 (0%)	0.01
*bla*CTX-M-15	14 (6%)	1 (2%)	0.21
*bla*CTX-M-3	9 (4%)	1 (2%)	0.69
*qnrS1*	68 (31%)	3 (5%)	<0.01
*qnrB4*	13 (6%)	4 (7%)	0.76
*gyrA*-83 mutation	34 (16%)	1 (2%)	<0.01
*parC*-80 mutation	26 (12%)	0 (0%)	<0.01
**Phenotypic antibiotic resistance**			
Extended-spectrum beta-lactamase phenotype	47 (22%)	5 (9%)	0.04
Ciprofloxacin	142 (66%)	26 (46%)	0.01
Cefotaxime	67 (31%)	10 (18%)	0.05
Ertapenem	2 (1%)	0 (0%)	1.00
Levofloxacin	68 (31%)	8 (14%)	0.01
Meropenem	2 (1%)	0 (0%)	1.00

^a^Continuous variables: mean (SD).

### ST23 was the commonest sequence type of Klebsiella pneumoniae

**Fig. 1:**
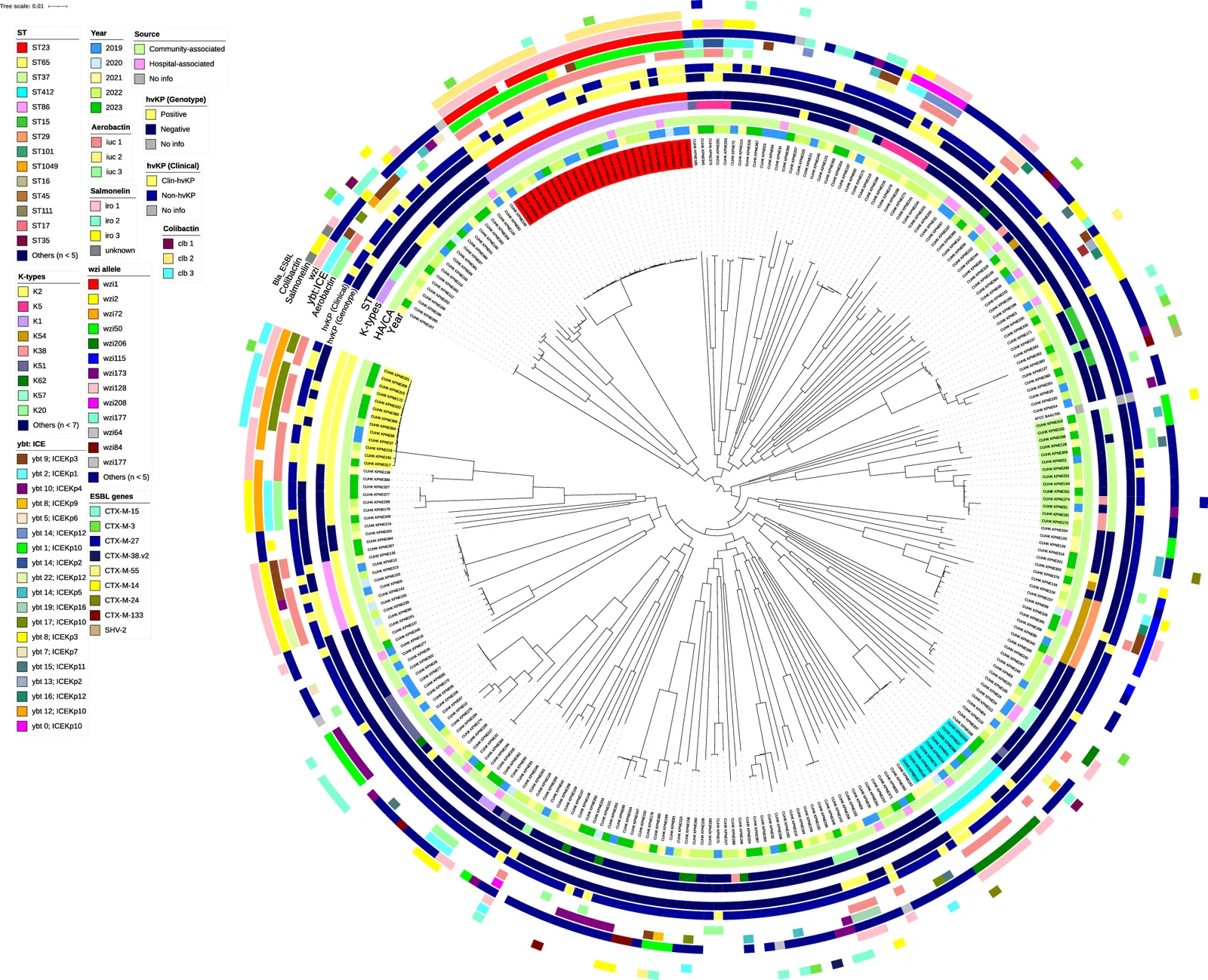
Phylogenetic tree of all 273 KP isolates. This figure illustrates the phylogenetic relationship of all KP isolates in the study, demonstrating the heterogeneity in ST, K-types, and virulence genes.

Figure 1 illustrates the phylogenetic analysis of all isolates in this study, with *Klebsiella pneumoniae* BAA-1705 used as the reference. The commonest ST was ST23 (*n* = 24, 8.8%), followed by ST65 (*n* = 13, 4.8%) and ST37 (*n* = 12, 4.4%), indicating the heterogeneity of *Klebsiella pneumoniae* causing invasive infections in this cohort. The majority of *Klebsiella pneumoniae* isolates were K2 type (*n* = 38, 13.9%), followed by K1 (*n* = 29, 10.6%). Among the K1 serotype, 20 of them (69%) were classified as g-hvKP. Among the K2 serotype, 14 isolates (37%) were classified as g-hvKP. All ST23 strains were K1, while all ST65 strains were K2.

Further sequence analysis showed that there were no deletions or protein-truncating variants in *iro*, *rmpA*, *rmpA2*, and *peg-344* of all isolates. Among all isolates with aerobactin (*iuc*), only 1 isolate bearing *iuc1* and 2 isolates bearing *iuc2* had frameshift variants compared to the reference genome.

### Resistance to cephalosporins and fluoroquinolones were less common in g-hvKP than cKP

Resistance was observed most commonly in fluoroquinolones (ciprofloxacin 61.5% and levofloxacin 27.8%), while resistance to beta-lactams was also noted (amoxicillin-clavulanate 30.4%, cefotaxime, 28.2%).

Chromosomal beta-lactamase *bla*SHV was found in 261 (95.6%) of isolates. For the remaining 12 isolates, 8 of them had truncated *bla*SHV. Forty-two (15.4%) isolates were found to harbour genes encoding extended-spectrum beta-lactamases (ESBL). The commonest types of which being were *bla*CTX-M-15 (*n* = 15, 5.5%) and *bla*CTX-M-3 (*n* = 10, 3.7%). Other common acquired beta-lactamases included *bla*DHA1 (*n* = 20, 7.3%), *bla*LAP2 (*n* = 24, 8.8%), and *bla*TEM1Dv1 (*n* = 40, 14.7%). Fifty-two isolates (19%) demonstrated the presence of ESBL on phenotypic testing.

Two isolates that were phenotypically resistant to ertapenem and meropenem: both harboured OmpK35/OmpK36 deletions. No isolates in this cohort carried any carbapenemase genes. Common fluoroquinolone resistance mechanisms were *qnrS1* (*n* = 71, 26.0%), *qnrB4* (*n* = 17, 6.2%), *gyrA*−83 mutation (*n* = 35, 12.8%), and *parC*−80 (*n* = 26, 9.5%) mutation.

g-hvKP were less resistant than cKP to beta-lactams and fluoroquinolones. Genotypically, this was shown by g-hvKP carrying fewer resistance genes (mean 0.7, SD 1.8) than cKP (mean 4.1, SD 5.0) (*p* < 0.01). Specifically, there was a lower rate of g-hvKP carrying *bla*TEM1Dv1 (*p* = 0.01), *bla*OXA (*p* = 0.01), *bla*LAP2 (*p* < 0.001), *gyrA*−83 mutation (*p* < 0.01), and *parC*−80 mutations (*p* < 0.01). The ESBL phenotype was more likely in cKP (*n* = 47, 22%) than g-hvKP (*n* = 5, 9%), which reached statistical significance (*p* = 0.04). The rate of *bla*ESBL was also lower in g-hvKP, but this did not reach statistical significance (*p* = 0.06). Phenotypically, the resistance rates of g-hvKP were lower than cKP for ciprofloxacin (*p* = 0.01), cefotaxime (*p* = 0.05), cefuroxime (*p* = 0.02), and levofloxacin (*p* = 0.01).

### GWAS reveals additional candidate genes for hypervirulent phenotype

**Fig. 2:**
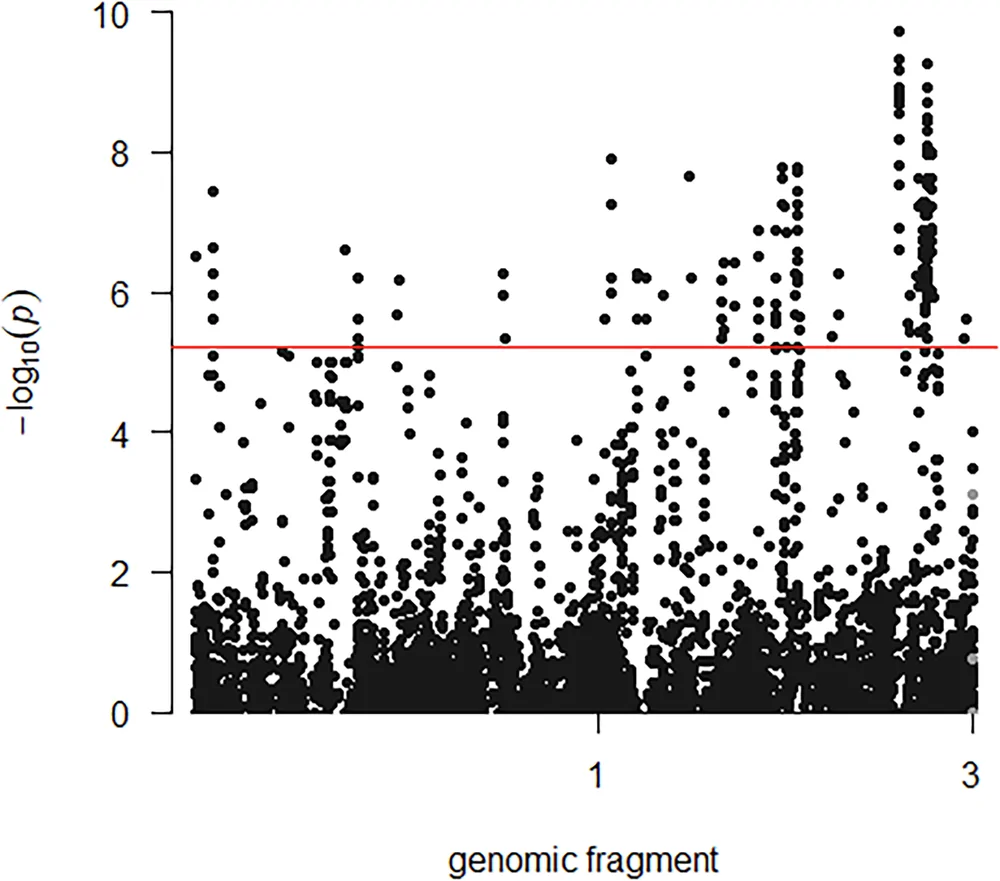
Manhattan plot of GWAS. This plot demonstrates the genetic association data across the genomic fragments. The horizontal red line represents the threshold of statistical significance by Bonferroni correction.

**Fig. 3:**
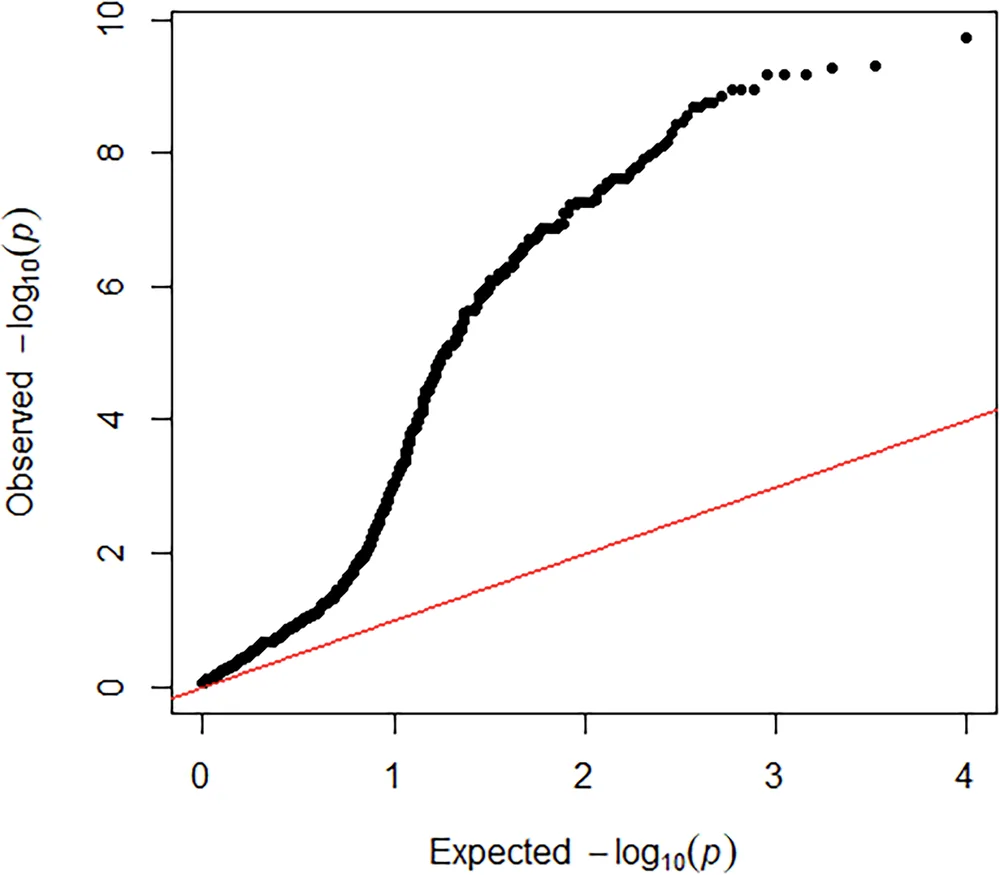
Q-Q plots of GWAS. This plot illustrates that the distribution of p-values in GWAS is not due to randomness.

Genome-wide association studies (GWAS) was carried out to analyse the association between the clinical hypervirulence phenotype and candidate genes of all isolates (*n* = 273). One hundred and five candidate genes showed significant association with the clinical hypervirulence phenotype. Based on Bonferroni correction, the *p*-value threshold was set at 6.07 × 10^–6^. The Manhattan plot (Fig. 2) and the Q-Q plot (Fig. 3) also showed that the observed distribution of *p*-values was not due to randomness. The candidate genes with the strongest associations with the clinical hvKP phenotype were *cysH3* (phosphoadenosine phosphosulfate reductase, OR = 7.53, *p* = 1.89E–10), *aidA* (qorum-quenching protein, OR = 7.15, *p* = 5.48E–10), and *pfeA2* (ferric enterobacin receptor, OR = 6.97, *p* = 6.89E–10). The top 12 candidate genes with *p*-values < 10^–^^8^ are illustrated in Table 2. The entire list of significant candidate genes is included in Supplementary Table 2. Using all potential combinations of the 3 genes with the strongest association as predictors, the NPV, PPV, specificity, and the sensitivity of these predictors were calculated. NPV was 89–90%, PPV was 45–48%, specificity was 79–82%, and sensitivity was 61–67%. The combination of all 3 markers had the best predictive power, showing higher NPV, PPV, and F1 score than the 5-gene definition or the genotypic determinants of hypermucoidy. The details of the predictive performance of these genes with the clinical phenotype is detailed in Table 3.

**Table 2:** Candidate genes in GWAS showing significant association with clinical hypervirulence phenotype

Gene	Gene annotation	*p*-value	Odds ratio
*cysH3*	Phosphoadenosine phosphosulfate reductase	1.89E–10	7.53
*aidA*	Quorum-quenching protein AidA	5.48E–10	7.15
*pfeA2*	Ferric enterobactin receptor	6.89E–10	6.97
*-*	IS5 family transposase IS903	1.16E–09	7.04
*btuD6*	Multidrug efflux ATP-binding/permease protein	1.16E–09	6.76
*fecR*	Protein FecR	1.75E–09	6.64
*decR2*	DNA-binding transcriptional activator DecR	1.98E–09	6.81
*fecI*	putative RNA polymerase sigma factor FecI	2.08E–09	6.73
*fes2*	Enterochelin esterase	2.70E–09	6.61
*pifC*	Transcriptional repressor PifC	3.32E–09	6.59
*-*	Tn3 family transposase ISYps3	3.81E–09	6.65
*ppsC2*	Alcohol dehydrogenase	4.85E–09	6.45

**Table 3:** Diagnostic performance of various genotypic determinants for detecting the clinical hvKP phenotype by various genotypic determinants

Genes, or combination of genes	TN	FN	FP	TP	NPV	PPV	Sp	Sn	F1
Existing genotypic determinants of hypervirulence and their permutations									
5-gene definition: *rmpA, rmpA2, iucA, iroA, peg-344*	185	31	31	26	86%	46%	86%	46%	0.46
*rmpA, rmpA2, iucA, peg-344* (no *iroA*)	185	31	31	26	86%	46%	86%	46%	0.46
*rmpA, rmpA2, iroA, peg-344* (no *iucA*)	185	31	31	26	86%	46%	86%	46%	0.46
*rmpA2, iucA, iroA, peg-344* (no *rmpA*)	185	31	31	26	86%	46%	86%	46%	0.46
*rmpA, rmpA2, iucA, iroA* (no *peg-344*)	185	31	31	26	86%	46%	86%	46%	0.46
*rmpA, iucA, iroA, peg-344* (no *rmpA2*)	166	50	20	37	77%	65%	89%	43%	0.51
Genotypic determinant of hypermucoidy									
Either *rmpA* or *rmpA2*	164	20	52	37	89%	42%	76%	65%	0.51
Kleborate virulence score cut-off									
VS ≥ 1	111	105	11	46	51%	81%	91%	30%	0.44
VS ≥ 2	150	66	16	41	69%	72%	90%	38%	0.50
VS ≥ 3	155	61	18	39	72%	68%	90%	39%	0.50
VS ≥ 4	172	44	26	31	80%	54%	87%	41%	0.47
VS ≥ 5	203	13	40	17	94%	30%	84%	57%	0.39
Alternative candidate genes from GWAS									
*cysH3*	171	19	45	38	90%	46%	79%	67%	0.54
*pfeA2*	171	20	45	37	90%	45%	79%	65%	0.53
*aidA*	177	22	39	35	89%	47%	82%	61%	0.53
Either *cysH3* or *pfeA2*	171	19	45	38	90%	46%	79%	67%	0.54
Either *pfeA2* or *aidA*	170	20	46	37	89%	45%	79%	65%	0.53
Either *cysH3* or *aidA*	170	19	46	38	90%	45%	79%	67%	0.54
Both *cysH3* and *pfeA2*	171	20	45	37	90%	45%	79%	65%	0.53
Both *pfeA2* and *aidA*	178	22	38	35	89%	48%	82%	61%	0.54
Both *cysH3* and *aidA*	178	22	38	35	89%	48%	82%	61%	0.54
At least 1 out of 3: *cysH3*, *pfeA2*, *aidA*	170	19	46	38	90%	45%	79%	67%	0.54
2 out of 3: *cysH3*, *pfeA2*, *aidA*	171	19	45	38	90%	46%	79%	67%	0.54
3 out of 3: *cysH3*, *pfeA2*, *aidA*	178	22	38	35	89%	48%	82%	61%	0.54

*TN* true negative, *FN* false negative, *FP* false positive, *TP* true positive.

*NPV* negative predictive value, *PPV* positive predictive value, *Sp* specificity, *Sn* sensitivity.

*F1* harmonic F1 score (F1 = 2 × Sn × PPV/(Sn + PPV)).

Using the isolates from 4 NCBI collections of *Klebsiella pneumoniae* as described in Methods section, invasive isolates were more likely to harbour the additional candidate genes *cysH3*, *pfeA2*, and/or *aidA* than non-invasive isolates. The odds ratio was 3.58 (*p* < 0.001). For details of gene distribution in the external collections, please refer to Supplementary Table 3.

### Clinical manifestations and association with virulence factors

The main differences of g-hvKP and cKP were found in the site of development, namely liver abscess and biliary tract infection, where the proportion of liver abscess was much higher in g-hvKP (*n* = 24, 42%) than cKP (*n* = 23, 11%) (*p* < 0.01). In contrast, the proportion of biliary tract infection was higher in isolates with cKP (*n* = 74, 34%) than g-hvKP (*n* = 11, 19%) (*p* = 0.04). For pneumonia, urinary tract infection, other intra-abdominal infection, bone and joint infection, endophthalmitis, and meningitis, the percentages between cKP and g-hvKP were not statistically significant. Fifty-six percent of hvKP bloodstream infection cases were due to sources other than liver abscess.

As for medical comorbidities, most patients were suffering from either diabetes mellitus (*n* = 166, 60.8%) or pre-diabetes (*n* = 54, 19.8%) regardless of g-hvKP or cKP. The proportion of patients suffering from hepatobiliary and/or pancreatic malignancy was higher in cKP (*n* = 41, 19%) than g-hvKP (*n* = 3, 5%) (*p* = 0.01).

### Various definitions of g-hvKP could not accurately predict the clinical phenotype of hvKP

Table 3 is the confusion matrix comparing the clinical definition of hvKP and various definitions of g-hvKP.

Using the clinical definition of hvKP as reference, the 5-gene definition of g-hvKP had a sensitivity of 46%, specificity of 86%, NPV of 86%, and PPV of 46%. In our cohort, the discriminatory power of the 5-gene definition (*iucA*, *iroB*, *rmpA*, *rmpA2*, *peg*-*344*) was identical to the 4-gene definitions by leaving out either *iucA*, *iroB*, *rmpA*, or *peg-344*.

However, when a 4-gene g-hvKP definition was constructed by leaving out *rmpA2*, the overall discriminatory power in terms of the F1 score improved to 0.51 from 0.46 of the 5-gene definition. Even so, the PPV and NPV of such definition were only 65% and 77% respectively.

Another definition of hypervirulence was constructed using the presence of genotypic determinants of hypermucoidy, i.e., the presence of either *rmpA* or *rmpA2*. This yielded an F1 score of 0.51, identical to that of the 4-gene definition without *rmpA2*. The PPV and NPV of this definition were 42% and 89%.

Finally, when specific cut-offs of Kleborate score were used for predicting c-hvKP, the best markers were the presence of *clb* (virulence score = 2, F1 score = 0.50) and the presence of *iuc* (virulence score = 3, F1 score = 0.50). The performance of these were similar to either the 4-gene definition leaving out *rmpA2* and the hypermucoidy definition.

### Overall mortality, 14-day mortality and disease severity were not associated with g-hvKP

**Fig. 4:**
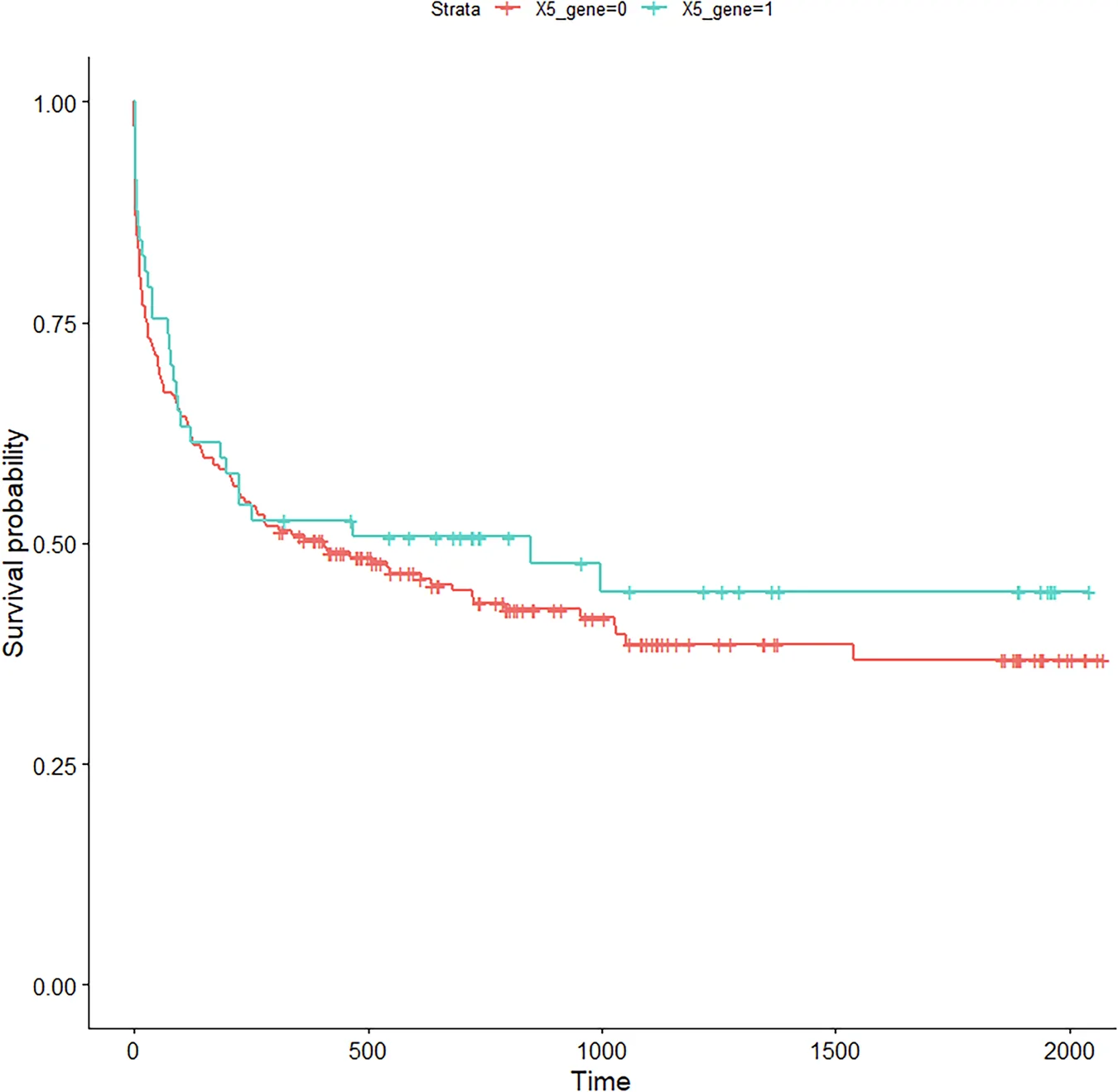
Survival curve of patients with cKP (red) and g-hvKP (green) infection. There was no statistically significant difference in patient survival between cKP and g-hvKP infections.

Figure 4 shows the survival curves of patients with cKP (red) and g-hvKP (green) infection. There was no statistically significant difference between survival in cKP and g-hvKP infections.

The key predictive factors of overall survival in Cox regression were ACCI > 5 (HR 3.29, *p* < 0.001), hepatobiliary cancer (HR 3.08, *p* < 0.001), creatinine > 250 µmol/L (HR 1.84, *p* = 0.01), pneumonia (HR 2.24, *p* < 0.001), inotrope use (HR 2.09, *p* < 0.001). g-hvKP was not a statistically significant predictor of mortality (HR 1.02, *p* = 0.938).

For 14-day mortality, the key predictive factors in multivariable logistic regression were ACCI > 5 (OR 2.63, *p* = 0.006), pneumonia (OR 4.52, *p* < 0.001), and inotrope use (OR 3.67, *p* < 0.001). g-hvKP was also not a statistically significant predictor of mortality (OR 0.54, *p* = 0.163).

The APACHE II scores of the cKP group (mean 26.7, SD 11.2) and the g-hvKP group (mean 22.7, SD 10.9) were not statistically significantly different (*p* = 0.36). Using the requirement for inotropic support within 1 day of specimen collection as a marker of disease severity, the key predictive factors in multivariable logistic regression were creatinine > 250 µmol/L (OR 3.36, *p* = 0.003) and colorectal carcinoma (OR 4.41, *p* = 0.004). g-hvKP was not a statistically significant predictor (OR 1.85, *p* = 0.077) of the use of inotrope in the multivariable model.

### Half of the liver abscess cases were caused by cKP

The development of liver abscess was strongly associated with g-hvKP (OR 6.87, *p* < 0.001) in multivariable logistic regression. While there was a specific association between liver abscess and g-hvKP, among the 47 cases of liver abscess in this cohort, only half (*n* = 24, 51%) were caused by g-hvKP. The other half (*n* = 23, 49%) of liver abscesses were caused by cKP. Comparing liver abscess cases in the cKP group and the g-hvKP group, more patients with cKP-related liver abscess had malignancy in the hepatobiliary-pancreatic system (*p* = 0.023). As for other medical comorbidities such as diabetes mellitus and gallstones, there was no statistically significant difference between cKP and g-hvKP. For a further detailed comparison of liver abscess due to cKP and g-hvKP, please refer to Supplementary Table 4.

### The development of metastatic infection, biliary tract infection, or pneumonia were not associated with g-hvKP in multivariable logistic regression

In multivariable logistic regression using the development of metastatic infection as outcome, the key predictive factor was inotrope use (OR 4.27, *p* = 0.002). Other factors, including g-hvKP (OR 2.06, *p* = 0.158) and number of classes of resistance genes (OR 0.86, *p* = 0.135), did not reach statistical significance.

Focusing on biliary tract infection, the statistically significant factors in multivariable logistic regression for the development of biliary tract infection included the presence of gallstones (OR 12.16, *p* < 0.001) andthe presence of hepatobiliary or pancreatic malignancy (OR 15.77, *p* < 0.001). g-hvKP was not associated with the development of biliary tract infection (OR 0.68, *p* 0.381).

As for pneumonia, the key predictive factors for the development of pneumonia were ACCI > 5 (OR 2.26, *p* = 0.040), resident of elderly care homes (OR 2.89, *p* = 0.019), history of cerebrovascular disease (OR 2.44, *p* = 0.025), and inotrope use (OR 3.37, *p* = 0.002). g-hvKP itself was not associated with pneumonia (OR 1.01, *p* = 0.988).

### Re-analysis using genotypic markers of hypermucoidy showed similar results

Using intact *rmpA* and/or *rmpA2* to define g-hvKP, the analysis as per sections 2.3 to 2.7 above was replicated. The sensitivity, specificity, NPV, and PPV of *rmpA*/*rmpA2* in predicting the clinical definition of hvKP were 65%, 76%, 89%, and 42% respectively. The presence of intact *rmpA* and/or *rmpA2* was not associated with overall mortality (HR 1.20, *p* = 0.938), 14-day mortality (OR 0.63, *p* = 0.223), inotrope use (OR 1.33, *p* = 0.358), metastatic infection (OR 1.74, *p* = 0.257), pneumonia (OR 0.75, *p* = 0.451), or biliary infection (OR 0.66, *p* = 0.265) in the respective multivariable regression models. On the other hand, liver abscess was associated with the genotypic markers of hypermucoidy (OR 8.97, *p* < 0.001).

## Discussion

The genotypes of strains in this cohort demonstrate validity with previous global studies on the genomic epidemiology of *Klebsiella pneumoniae*. The 2 commonest STs in this study were ST23 and ST65, both of which were commonly found in previous global studies^20^.

Our study showed a similar rate of nosocomial infection in cKP and g-hvKP. While the classical description of hvKP infection is of community onset, recent studies showed the emergence of nosocomial hvKP infection^21,22^.

The emergence of carbapenemase-producing hvKP has been noted in other regions of the world^6^. A global study noted an ESBL prevalence of 48% and carbapenemase prevalence of 43%^20^. In contrast, none of the strains in our cohort carried any carbapenemase genes. blaESBL was found in 15.4% (*n* = 42) of isolates, proving that the burden of antimicrobial resistance in *Klebsiella pneumoniae* in our study was not as high as in the other regions.

The authors noted a similar study on molecular epidemiology and clinical impact of *Klebsiella* spp. bacteraemia in Hong Kong in the period 2009–2018^16^. The serotype distribution was similar to that of our study, showing a predominance of K1 and K2 serotypes. The prevalences of various genotypic determinants of hypervirulence were similar between our study and their study. The proportions of ESBL isolates were also similar between the two studies. The higher mortality in their study could be attributed to various reasons such as case selection and change in medical practice over the years. In their study, markers of hypervirulence including the presence of hypervirulence or a positive string test were also not associated in any difference in mortality. While we did not perform string test in our study, we analysed the effect of either *rmpA* or *rmpA2* as the genotypic marker of hypermucoidy in our statistical analysis. Other studies in China and Korea noted more community-onset infections and lower mortality with hvKP^23,24^. Furthermore, some researchers showed that among patients with hvKP infection, ICU admission and salmochelin carriage were associated with in-hospital mortality^25^. The epidemiology and the background of these studies were similar to ours. Some of the differences in findings can be attributed to changes in epidemiology over time and the use of different definitions for hvKP. Interestingly, inappropriate empirical antibiotics were not a predictor of mortality in our study, in contrast to a previous study performed in our population^26^.

The GWAS result revealed additional candidate genes which may help us further understand the pathogenesis of the observed clinical hypervirulence phenotype and more accurately define the genotypic determinants of hvKP. Out of the 12 significant candidate genes, 5 were related to iron transport and metabolism (*pfeA*, IS903, *fecR*, *fecI*, *fes2*)^27–32^, and 2 were related to sulfur metabolism (*cysH* and *decR*)^33,34^. Superior iron acquisition has been described repeatedly as a hallmark of hvKP^35–37^. Iron-sulfur clusters are also essential in iron uptake, capsular polysaccharide synthesis, and overcoming colonisation resistance^38–40^. Using this set of genomic markers, the diagnostic performance of clinical hvKP phenotype was better than the previous 5-gene definition or the genotypic markers of hypermucoidy. This alternative set of markers had better NPV, sensitivity, and harmonic F1 score than the previous definitions. Using 4 well-annotated collections from NCBI from the USA and Hong Kong, invasive isolates were more like to have one or more candidate genes than non-invasive isolates. However, further studies in characterisation of these genes in causing the hvKP phenotype using a global database is required before further generalisation.

This study calls for a revision of the understanding of the disease phenotype of hvKP. We found significant overlap in the disease spectrum of cKP and g-hvKP in our cohort using a variety of genotypic markers of hypervirulence. Only half of liver abscess cases (51%) in this cohort were caused by g-hvKP. There was no statistical significance when comparing cKP and g-hvKP on other disease phenotypes. There were no significant differences in the rate of community-onset infection or proportion of care home residents between cKP and g-hvKP. There was also no difference in key clinical outcomes such as disease severity and mortality between the two groups. These findings echo the call for more prudent use of the term “hypervirulence” in the literature when describing *Klebsiella pneumoniae*^41^. We would therefore like to propose that subsequent attempts at any genotypic definition of “hypervirulent” *Klebsiella pneumoniae* be firmly rooted in the human clinical manifestations rather than in vitro or in silico data.

Our study contradicts the conclusion of a previous study showing a difference in 14-day mortality using a 5-gene definition for g-hvKP^11^, as well as another study that showed the superiority of a 5-gene definition over other definitions of g-hvKP^10^. In contrast to our study which utilised Cox regression, the survival outcomes tested by Tang et al., 2025 was performed using repeated testing on consecutive time-points at 14 days, 28 days, and 90 days^11^. The *p*-values obtained from that analysis after propensity score matching were 0.032, 0.071, and 0.551 respectively as per Table 2 of the study^11^. After the application of Bonferroni correction for repeated testing, none of their *p*-values were significant as all were larger than 0.013 (0.05 divided by 3). This indicated that patient survival did not differ between g-hvKP and cKP if more robust statistical testing were implemented in that study. Another contrast with that study was the actual clinical manifestation of the patients. In our study, the medical record of each patient was examined to determine the type of infection for each case. In that study, the type of the clinical specimen of the isolate was only briefly mentioned where no reference to the infection syndrome was disclosed^11^. For instance, some isolates in the said study were labelled as “bloodstream” or “abdominal cavity”^11^. From a clinical point of view, this is not informative in differentiating whether the clinical phenotype was due to cKP or hvKP. Both liver abscess (a “hypervirulent” phenotype) and cholangitis (a “classical” phenotype) can result in bacteraemia (i.e., an isolate from bloodstream). Isolates from the abdominal cavity can be due to perforated appendicitis (a “classical” phenotype) or liver abscess (a “hypervirulent” phenotype). Therefore, no comparison between the definition of g-hvKP and actual clinical manifestation was able to be made in the said study^11^. Therefore, apart from replicating the analysis on survival, our study additionally showcased a detailed analysis of the correlation between various genotypic markers against an array of clinical manifestations. Furthermore, we demonstrated that a 4-gene definition without *rmpA2* gene performed better than the originally proposed 5-gene definition in terms of harmonic F1 score, with that of the former being 0.51 and that of the latter being 0.46.

Given the significant overlap in clinical phenotype between cKP and hvKP as demonstrated in our study using a variety of well-known genotypic markers, we would also like to propose that *Klebsiella pneumoniae* infection be considered more broadly. We acknowledge that some markers may point towards a higher incidence of a specific manifestation, for instance the association between *iuc* and liver abscess in our study. However, such link is not absolute and using such markers to delineate clinical disease manifestation is potentially misleading. As shown in our study, the sensitivity and the specificity of the 5-gene definition in detecting the clinical phenotype of hvKP were only 46% and 86% respectively. Therefore, when treating patients with invasive *Klebsiella pneumoniae* infection, it would be more prudent to consider this entity as a whole rather than emphasising the difference between cKP and hvKP.

One limitation of our study is that only patients with bacteraemia or meningitis were included. Other specimen sites, including wound swabs, pus, urine, or sputum, were not included. However, isolation of organisms from latter specimens may represent colonisation, diluting the phenotypical virulence of genuine invasive infections found in this study.

Another limitation is related to the definition of hypermucoidy. Hypermucoidy is a complex phenomenon regulated by *rmpA*, *rmpA2*, *rmpC*, *rmpD*, *wzc* mutations, and other genetic and environmental determinants. *rmpA* and *rmpA2* are two of the potential combinations that incompletely characterise this phenomenon. To confirm the phenotype of hypermucoidy, capsule quantification would be a more appropriate phenotypic test. This was not performed within the setting of our study but would form one of the future directions of this research theme.

A further limitation is the definition of hvKP in this study. We note that a variety of markers have been included in previous studies, with varying sensitivity and specificity. The inclusion of both the 5-gene definition and the genotypic markers of hypermucoidy in our analysis was an effort to ensure the robustness of the findings. Furthermore, phenotypic assays of hypervirulence such as murine models, siderophore detection were not performed in our study. In vitro phenotypic studies showed suboptimal correlation between phenotypic assays and genotypic traits of hypervirulence^18,42^. A further direction would be to include data of phenotypic assays to further enrich our dataset.

The genomic markers identified from GWAS did not undergo any in-depth elaboration, experimental validation, or analysis of their molecular characteristics. Therefore, it is imperative that further research be performed before these markers can be implemented in the clinical setting. A future direction is to perform experimental validation on these alternative markers.

Given the low rate of meningitis and endophthalmitis, the lack of statistical significance in the comparison between cKP and g-hvKP in this aspect may be due to the low number of events. A medium-term series focusing on nervous system infections may help clarify this issue.

## Conclusion

There was significant overlap in disease phenotype between cKP and g-hvKP, ranging from liver abscess to other manifestations. g-hvKP was not associated with mortality or disease severity.

## Supplementary information

Supplementary Information

Description of Additional Supplementary Files

Supplementary Data 1

Supplementary Data 2

Supplementary Data 3

## Data Availability

This study is performed on clinical dataset and sequencing dataset. All relevant clinical data are available from the corresponding author upon request. Genomes of all isolates were deposited on NCBI GeneBank (accession number: PRJNA1307753). Figure source data can be found as Supplementary Data.
