# Supplementary material for: Revisiting definitions of hypervirulent and classical Klebsiella pneumoniae through virulence factors and disease phenotype
Source: Commun Med (Lond). 2026 May 27;6:450. doi: 10.1038/s43856-026-01613-7 (PMC13493853; doi:10.1038/s43856-026-01613-7)
Supplement: Supplementary file 1 [file 43856_2026_1613_MOESM1_ESM.pdf]

Supplementary Table 1. Epidemiological, clinical, and genomic comparison of cKP and g-hvKP

Variables	cKP (n=216) Count (%)	g-hvKP (n=57) Count (%)	p-values
Epidemiological characteristics			
Age in years*	72.4 (14.6)	71.4 (15.2)	0.75
Male gender	127 (59%)	29 (51%)	0.30
Hospital-onset infection	20 (9%)	3 (5%)	0.43
Residents of elderly care home	30 (14%)	14 (25%)	0.07
14-day mortality	45 (21%)	9 (16%)	0.46
30-day mortality	58 (27%)	12 (21%)	0.40
1-year mortality	107 (50%)	27 (47%)	0.88
Clinical types of infection			
Metastatic infection (2 or more sites involved)	12 (6%)	9 (19%)	0.02
Liver abscess	23 (11%)	24 (42%)	<0.01
Biliary infection	74 (34%)	11 (19%)	0.04
Other intra-abdominal infection	12 (6%)	1 (2%)	0.31
Pneumonia	38 (18%)	13 (23%)	0.44
Urinary tract infection	52 (24%)	9 (16%)	0.21
Bone and joint infection	4 (2%)	1 (2%)	1.00
Central nervous system infection	1 (0%)	1 (2%)	0.37
Endophthalmitis	1 (0%)	2 (4%)	0.11
Severity of infection and clinical management			
APACHE II score*	26.7 (11.2)	22.7 (10.9)	0.36
Intensive care unit admission	22 (10%)	11 (19%)	0.07
Inotrope use	43 (20%)	17 (30%)	0.15
Effective antibiotics within 48 hours of onset	194 (90%)	51 (89%)	1
Medical comorbidities			
Age-adjusted Charlson comorbidity index*	5.8 (3)	4.9 (3.1)	0.05
Diabetes mellitus	132 (61%)	34 (60%)	0.88
Pre-diabetes	41 (19%)	13 (23%)	0.58
Hepatitis C virus infection	0 (0%)	1 (2%)	0.21
Hepatitis B virus chronic infection	19 (9%)	6 (11%)	0.62
Immunosuppressant use	43 (20%)	8 (14%)	0.35
History of malignancy	13 (6%)	3 (5%)	1.00
Colorectal carcinoma	14 (6%)	3 (5%)	1.00
Hepatobiliary or pancreatic malignancy	42 (19%)	3 (5%)	0.01
Other solid organ malignancy	14 (6%)	3 (5%)	1.00
Haematological malignancy	11 (5%)	0 (0%)	0.13
Cirrhosis	12 (6%)	4 (7%)	0.75
Gallstones	52 (24%)	9 (16%)	0.21
Myocardial infarction	11 (5%)	3 (5%)	1.00
Congestive heart failure	18 (8%)	6 (11%)	0.60
Peripheral vascular disease	3 (1%)	0 (0%)	1.00
Cerebrovascular disease	50 (23%)	12 (21%)	0.86

Dementia	8 (4%)	1 (2%)	0.69
Chronic pulmonary disease	19 (9%)	3 (5%)	0.58
Rheumatic disease	0 (0%)	0 (0%)	1.00
Peptic ulcer disease	22 (10%)	4 (7%)	0.62
Renal disease	29 (13%)	4 (7%)	0.25
Hypertension	84 (39%)	23 (40%)	0.88
Blood test results			
Alanine aminotransferase (IU/mL)*	73.4 (92.8)	80.6 (76.3)	0.06
Alkaline phosphatase (IU/mL)*	213.8 (188.7)	176.2 (148.7)	0.27
Bilirubin (μmol/L)*	40.6 (56.9)	30 (32.3)	0.65
Creatinine (μmol/L)*	168.6 (202.5)	134.9 (97.3)	0.55
Haemoglobin (g/dL)*	10.8 (2.5)	10.7 (2.5)	0.91
ANC (10^9/L)*	12.5 (9.4)	11.5 (6.7)	0.95
WBC (10^9/L)*	13.8 (9.8)	12.8 (6.6)	0.89
Genotypic epidemiology			
ST23	4 (2%)	20 (35%)	<0.01
ST65	6 (3%)	7 (12%)	0.01
ST37	12 (6%)	0 (0%)	0.08
K1 serotype	9 (4%)	20 (35%)	<0.01
K2 serotype	24 (11%)	14 (25%)	0.02
O1O2v1 serotype	74 (34%)	16 (28%)	0.43
O1O2v2 serotype	70 (32%)	31 (54%)	<0.01
Genotypic markers of virulence			
ybt	84 (39%)	42 (74%)	<0.01
clb	13 (6%)	24 (42%)	<0.01
iucA	43 (20%)	57 (100%)	<0.01
iroB	44 (20%)	57 (100%)	<0.01
rmpA	44 (20%)	57 (100%)	<0.01
rmpA2	1 (0%)	57 (100%)	<0.01
peg-344	44 (20%)	57 (100%)	<0.01
KpVP-1 virulence plasmid	19 (8%)	56 (98%)	<0.01
KpVP-2 virulence plasmid	6 (3%)	0 (0%)	0.59
Genotypic markers of resistance			
Number of resistance genes*	4.1 (5.0)	0.7 (1.8)	<0.01
DHA1	15 (7%)	5 (9%)	0.58
LAP2	24 (11%)	0 (0%)	<0.01
TEM1Dv1	38 (18%)	2 (4%)	0.01
OXA	23 (11%)	0 (0%)	0.01
Genes encoding extended-spectrum beta-lactamases	38 (18%)	4 (7%)	0.06
CTXM15	14 (6%)	1 (2%)	0.21
CTXM3	9 (4%)	1 (2%)	0.69
qnrS1	68 (31%)	3 (5%)	<0.01
qnrB4	13 (6%)	4 (7%)	0.76
gyrA83	34 (16%)	1 (2%)	<0.01

parC80	26 (12%)	0 (0%)	<0.01
Phenotypic antibiotic resistance			
Extended-spectrum beta-lactamase	47 (22%)	5 (9%)	0.04
Amikacin	3 (1%)	0 (0%)	1.00
Amoxicillin-clavulanate	71 (33%)	12 (21%)	0.11
Cefepime	32 (15%)	5 (9%)	0.28
Ciprofloxacin	142 (66%)	26 (46%)	0.01
Cefotaxime	67 (31%)	10 (18%)	0.05
Cefuroxime	79 (37%)	11 (19%)	0.02
Ertapenem	2 (1%)	0 (0%)	1.00
Gentamicin	19 (9%)	1 (2%)	0.09
Levofloxacin	68 (31%)	8 (14%)	0.01
Meropenem	2 (1%)	0 (0%)	1.00
Piperacillin-tazobactam	70 (32%)	13 (23%)	0.20
*Continuous variables: mean (SD)			

Supplementary Table 2. Full list of genes with significant association with the clinical hypervirulence phenotype

Gene	Annotation	p-value	Odds ratio
cysH_3	Phosphoadenosine phosphosulfate reductase	1.89E-10	7.53
aidA	Quorum-quenching protein AidA	5.48E-10	7.15
pfeA_2	Ferric enterobactin receptor	6.89E-10	6.97
-	IS5 family transposase IS903	1.16E-09	7.04
btuD_6	Multidrug efflux ATP-binding/permease protein	1.16E-09	6.76
fecR	Protein FecR	1.75E-09	6.64
decR_2	DNA-binding transcriptional activator DecR	1.98E-09	6.81
fecI	putative RNA polymerase sigma factor FecI	2.08E-09	6.73
fes_2	Enterochelin esterase	2.70E-09	6.61
pifC	Transcriptional repressor PifC	3.32E-09	6.59
-	Tn3 family transposase ISYps3	3.81E-09	6.65
ppsC_2	Alcohol dehydrogenase	4.85E-09	6.45
-	Tn3 family transposase ISNpu13	1.11E-08	6.19
-	IS1 family transposase IS1N	1.50E-08	6.00
iprA_1	Inhibitor of hydrogen peroxide resistance	2.18E-08	11.83
def_1	Peptide deformylase	2.43E-08	5.82
xerC_2	Tyrosine recombinase XerC	2.43E-08	5.82
ybdM_2	putative protein YbdM	2.84E-08	5.90
iucB	N(6)-hydroxylysine O-acetyltransferase	5.02E-08	5.59
iucD	L-lysine N6-monooxygenase	5.02E-08	5.59
ptsJ_2	Vitamin B6 salvage pathway transcriptional repressor PtsJ	5.33E-08	6.24
-	IS3 family transposase IS1400	5.56E-08	13.47
-	IS3 family transposase IS1400	5.56E-08	13.47
iutA_2	Ferric aerobactin receptor	5.66E-08	5.47
-	IS3 family transposase ISSen4	6.10E-08	5.65
-	IS21 family transposase ISSen8	6.10E-08	5.65
nikA_2	Nickel-binding protein NikA	8.22E-08	5.54
repB_1	RepFIB replication protein A	8.22E-08	5.54
noc	Nucleoid occlusion protein	1.19E-07	5.71
oppF_4	Oligopeptide transport ATP-binding protein OppF	1.30E-07	5.42
oppD_3	Oligopeptide transport ATP-binding protein OppD	1.30E-07	5.42
-	putative oxidoreductase/MSMEI_2347	1.30E-07	5.42
-	IS3 family transposase ISEc27	1.30E-07	5.42
-	IS3 family transposase IS1222	1.30E-07	5.42
ybcJ	putative protein YbcJ	1.33E-07	9.39
ybcI	Inner membrane protein YbcI	1.33E-07	9.39
ybcJ	putative protein YbcJ	1.33E-07	0.11
-	IS3 family transposase ISYps8	1.44E-07	5.70
btuD_8	Vitamin B12 import ATP-binding protein BtuD	1.68E-07	5.05
-	IS110 family transposase IS1663	1.88E-07	6.84
thiL_2	Thiamine-monophosphate kinase	1.91E-07	5.82

nitA	Aliphatic nitrilase	1.91E-07	5.82
acsA	Acetyl-coenzyme A synthetase	1.91E-07	5.82
-	putative protein	2.31E-07	9.65
elmGT	Elloramycin glycosyltransferase ElmGT	2.47E-07	5.01
hemB_2	Delta-aminolevulinic acid dehydratase	2.73E-07	5.21
benC	Benzoate 1,2-dioxygenase electron transfer component	2.98E-07	8.50
iucA	N(2)-citryl-N(6)-acetyl-N(6)-hydroxylysine synthase	3.00E-07	4.89
vapC_2	tRNA(fMet)-specific endonuclease VapC	3.52E-07	5.05
-	IS3 family transposase IS1400	3.93E-07	10.02
iucC	Aerobactin synthase	4.77E-07	4.74
intS_1	Prophage integrase IntS	5.30E-07	8.65
gmm	GDP-mannose mannosyl hydrolase	5.30E-07	8.65
alx_2	Putative membrane-bound redox modulator Alx	5.62E-07	4.87
silE_2	Silver-binding protein SilE	5.68E-07	5.01
yciC_2	Putative metal chaperone YciC	5.68E-07	5.01
mdtH_3	Multidrug resistance protein MdtH	5.96E-07	5.33
gtfA	UDP-N-acetylglucosamine--peptide N-acetylglucosaminyltransferase GtfA subunit	6.33E-07	7.76
luxA_1	Alkanal monooxygenase alpha chain	6.33E-07	7.76
wzc	Tyrosine-protein kinase wzc	6.33E-07	7.76
glxR	2-hydroxy-3-oxopropionate reductase	6.91E-07	7.14
mdaB_2	NADPH:quinone oxidoreductase MdaB	6.91E-07	7.14
zur_2	Zinc uptake regulation protein	7.43E-07	4.85
yceD_2	General stress protein 16U	7.93E-07	4.63
yceD_3	General stress protein 16U	7.93E-07	4.63
stiP	Cysteine protease StiP	7.93E-07	4.63
yceC	Stress response protein SCP2	7.93E-07	4.63
-	ISNCY family transposase ISSen7	8.28E-07	4.70
yceD_4	General stress protein 16U	9.91E-07	4.63
higB	mRNA interferase HigB	1.10E-06	5.79
allS_1	HTH-type transcriptional activator AllS	1.33E-06	6.63
allD	Ureidoglycolate dehydrogenase (NAD(+))	1.33E-06	6.63
allE	(S)-ureidoglycine aminohydrolase	1.33E-06	6.63
gcl	Glyoxylate carboligase	1.33E-06	6.63
allR	HTH-type transcriptional repressor AllR	1.33E-06	6.63
ybbW	Putative allantoin permease	1.33E-06	6.63
-	IS110 family transposase ISKpn42	1.46E-06	4.41
qseB	Transcriptional regulatory protein QseB	2.14E-06	0.18
lspA_2	Lipoprotein signal peptidase	2.29E-06	4.79
hin	DNA-invertase hin	2.29E-06	4.79
nodD2_2	Nodulation protein D 2	2.34E-06	7.15
stp_2	Multidrug resistance protein Stp	2.34E-06	7.15
arsH	NADPH-dependent quinone reductase ArsH	2.34E-06	7.15
hpxO_2	FAD-dependent urate hydroxylase	2.34E-06	7.15
cbdA_2	2-halobenzoate 1,2-dioxygenase large subunit	2.34E-06	7.15

betA_1	Oxygen-dependent choline dehydrogenase	2.34E-06	7.15
rutC_1	Putative aminoacrylate peracid reductase RutC	2.34E-06	0.14
glxK	Glycerate 3-kinase	2.48E-06	6.19
arcC2	Carbamate kinase 2	2.48E-06	6.19
fdrA	Protein FdrA	2.48E-06	6.19
allA	Ureidoglycolate lyase	2.48E-06	6.19
sauU	putative sulfoacetate transporter SauU	2.49E-06	6.59
allC	Allantoate amidohydrolase	2.49E-06	6.59
fecA_2	Fe(3+) dicitrate transport protein FecA	2.79E-06	4.66
kdpB_2	Potassium-transporting ATPase ATP-binding subunit	3.39E-06	4.62
higA	Antitoxin HigA	3.66E-06	5.37
bglT	6-phospho-beta-glucosidase BglT	4.21E-06	7.20
rutC_2	Putative aminoacrylate peracid reductase RutC	4.47E-06	5.80
-	2,6-dihydropseudooxynicotine hydrolase	4.58E-06	6.57
hyi_2	Hydroxypyruvate isomerase	4.58E-06	6.57
kynB	Kynurenine formamidase	4.58E-06	6.57
ybbY	Putative purine permease YbbY	4.64E-06	6.13
allB_1	Allantoinase	4.64E-06	6.13
hyi_1	Hydroxypyruvate isomerase	4.64E-06	6.13
ccmM	Carbon dioxide concentrating mechanism protein CcmM	5.92E-06	4.10

Supplementary Table 3. Distribution of number of new candidate genes in 4 NCBI collections

pfeA2, cysH3, and/or *aidA* present?	Non-invasive isolates	Invasive isolates
None present	263	283
1 gene present	21	23
2 genes present	5	16
3 genes present	1	65

Chi-squared test was calculated based on:

pfeA2, cysH3, and/or *aidA* present?	Non-invasive isolates	Invasive isolates
None present	263	283
1 or more gene present	27	104

Supplementary Table 4. Comparison of cKP liver abscess and g-hvKP liver abscess

	cKP n = 23	g-hvKP n =24	P-values
Medical comorbidities			
Diabetes mellitus	13 (57%)	10 (42%)	0.387
Pre-diabetes	6 (27%)	8 (34%)	0.752
HCV infection	0 (0%)	1 (5%)	1
HBV infection	4 (18%)	1 (5%)	0.188
Immunosuppressant	6 (27%)	1 (5%)	0.090
Past malignancy	0 (0%)	0 (0%)	1
Colorectal carcinoma	0 (0%)	0 (0%)	1
Hepatobiliary/pancreatic carcinoma	7 (31%)	1 (5%)	0.023
Other solid organ cancer	0 (0%)	0 (0%)	1
Haematological malignancy	1 (5%)	0 (0%)	1
Cirrhosis	4 (18%)	1 (5%)	0.188
Gallstones	3 (14%)	3 (13%)	1
Myocardial infarction	1 (5%)	0 (0%)	1
Congestive heart failure	2 (9%)	2 (9%)	1
Peripheral vascular disease	0 (0%)	0 (0%)	1
Cerebrovascular disease	2 (9%)	4 (17%)	0.666
Dementia	1 (5%)	0 (0%)	1
Chronic pulmonary disease	4 (18%)	1 (5%)	0.188
Rheumatic disease	0 (0%)	0 (0%)	1
Peptic ulcer disease	5 (22%)	0 (0%)	0.022
Renal disease	2 (9%)	2 (9%)	1
Hypertension	4 (18%)	7 (30%)	0.494
Epidemiological markers			
K1	4 (18%)	12 (50%)	0.031
K2	7 (31%)	5 (21%)	0.517
O1O2v1	9 (40%)	7 (30%)	0.547
O1O2v2	10 (44%)	17 (71%)	0.080
Virulence genes			
ybt	14 (61%)	20 (84%)	0.036
clb	5 (22%)	13 (55%)	0.036
iuc	11 (48%)	24 (100%)	<0.001
iro	13 (57%)	24 (100%)	<0.001
rmpA	13 (57%)	24 (100%)	<0.001
rmpA2	0 (0%)	24 (100%)	<0.001
peg344	13 (57%)	24 (100%)	<0.001
