# Supplementary material for: Revisiting definitions of hypervirulent and classical Klebsiella pneumoniae through virulence factors and disease phenotype
Source: Commun Med (Lond). 2026 May 27;6:450. doi: 10.1038/s43856-026-01613-7 (PMC13493853; doi:10.1038/s43856-026-01613-7)
Supplement: Supplementary file 2 [file 43856_2026_1613_MOESM2_ESM.pdf]

Description of Additional Supplementary Files

File name: Supplementary Data 1

Description: Source data for Figure 1

File name: Supplementary Data 2

Description: Source data for Figure 2

File name: Supplementary Data 3

Description: Source data for Figure 3 & 4
